# A Novel Rat Model of Vitamin D Deficiency: Safe and Rapid Induction of Vitamin D and Calcitriol Deficiency without Hyperparathyroidism

**DOI:** 10.1155/2015/604275

**Published:** 2015-02-28

**Authors:** Andrea W. D. Stavenuiter, Maria Vittoria Arcidiacono, Evelina Ferrantelli, Eelco D. Keuning, Marc Vila Cuenca, Piet M. ter Wee, Robert H. J. Beelen, Marc G. Vervloet, Adriana S. Dusso

**Affiliations:** ^1^Department of Molecular Cell Biology & Immunology, VU University Medical Center, 1081 BT Amsterdam, Netherlands; ^2^Division of Experimental Nephrology, IRBLleida, 25198 Lleida, Spain; ^3^Department of Nephrology, VU University Medical Center, 1007 MB Amsterdam, Netherlands

## Abstract

Vitamin D deficiency is associated with a range of clinical disorders. To study the mechanisms involved and improve treatments, animal models are tremendously useful. Current vitamin D deficient rat models have important practical limitations, including time requirements when using, exclusively, a vitamin D deficient diet. More importantly, induction of hypovitaminosis D causes significant fluctuations in parathyroid hormone (PTH) and mineral levels, complicating the interpretation of study results. To overcome these shortcomings, we report the successful induction of vitamin D deficiency within three weeks, with stable serum PTH and minerals levels, in Wistar rats. We incorporated two additional manoeuvres compared to a conventional diet. Firstly, the vitamin D depleted diet is calcium (Ca) enriched, to attenuate the development of secondary hyperparathyroidism. Secondly, six intraperitoneal injections of paricalcitol during the first two weeks are given to induce the rapid degradation of circulating vitamin D metabolites. After three weeks, serum 25-hydroxyvitamin D_3_ (25D) and 1,25-dihydroxyvitamin D_3_ (1,25D) levels had dropped below detection limits, with unchanged serum PTH, Ca, and phosphate (P) levels. Therefore, this model provides a useful tool to examine the sole effect of hypovitaminosis D, in a wide range of research settings, without confounding changes in PTH, Ca, and P.

## 1. Introduction

Vitamin D deficiency is a worldwide problem [1] as it associates with increased mortality for all causes. In the US adult population, 42% is vitamin D deficient (25D serum levels <50 nmol/L; <20 ng/mL) [2]. Due to the small content of vitamin D in nonsupplemented food, circulating vitamin D levels are mainly determined by the amount of sunlight exposure. However, the high prevalence of vitamin D deficiency in the otherwise healthy US population varies not only depending upon seasonal changes, but also depending on cultural and racial factors. Cultural factors that limit skin exposure to sun light are clothing, age, sex, and the use of sunscreen protectors. Racial factors include a darker skin pigmentation, which limits conversion rates in spite of similar exposure to sun light [1–4]. The high incidence of obesity in the US population is an additional determinant of vitamin D deficiency, as circulating levels are lower due to dilution in the fat mass [5].

The epidemiological association between vitamin D deficiency and high mortality rates extends beyond the abnormal mineral homeostasis that impairs bone mineralization due to high serum levels of PTH. In fact, vitamin D deficiency associates with an increased risk of hypertension, cardiovascular disease, infectious and autoimmune diseases, glucose intolerance, albuminuria, and cancer [6–11]. Clearly, adequate strategies for vitamin D supplementation could help attenuate the incidence of these disorders, as suggested by the recent meta-analysis on the efficacy of cholecalciferol (vitamin D_3_), but not ergocalciferol (vitamin D_2_), supplementation in reducing mortality rates [12–14].

These observations urge for the need of animal models to study the mechanistic link between vitamin D deficiency and these clinical conditions. The most common models of vitamin D deficiency using a dietary approach, a vitamin D deficient diet, not only are time consuming and thus costly, but also have major limitations for result interpretation, specifically, due to the abnormalities in serum PTH, Ca, and P levels, which develop from the prolonged time required to achieve vitamin D deficiency due to the half-life of 15–18 days of serum 25D [15, 16]. In addition, these strategies may fail to achieve calcitriol deficiency in animals with normal kidney function because serum calcitriol levels decrease below the normal range only when serum 25D levels are lower than 4 ng/mL, that is, exclusively with severe vitamin D deficiency [17].

Herein, two important modifications to the most common dietary approach were used to achieve vitamin D and calcitriol deficiency within three weeks, while preventing increases in PTH and abnormal serum Ca and P levels. The first was the induction of renal CYP24A1, the enzyme responsible for the degradation of endogenous 25D and calcitriol, by administering the calcitriol analogue, paricalcitol. The second was the maintenance of normal intestinal P and Ca absorption and consequently normal serum PTH, through feeding the rats a high Ca, P, and lactose diet, similar to the rescue diet utilized in the vitamin D receptor (VDR) null mice. This diet ensures vitamin D independent intestinal Ca and P absorption [18, 19].

## 2. Methods

### 2.1. Animals and Experimental Design

Ten male Wistar rats (Harlan CPB, Horst, Netherlands), aged 9-10 weeks, weighing 300–325 grams at the beginning of the study were used. The rats underwent one-week acclimatization to the animal facility prior to the induction of vitamin D deficiency. Animals were maintained under conventional housing conditions and were given food and water* ad libitum*. From day one of the experiment, the rats were fed a vitamin D deficient diet (TD.87095 Brown C.C. Vit.D Defic, containing 20% lactose, 2% Ca, and 1.25% P). To induce CYP24A1 expression, to accelerate the catabolism of endogenous 25D and calcitriol stores, the rats received intraperitoneal injections of 32 ng of 19-nor-1,25-dihydroxyvitamin D2 (paricalcitol; Zemplar, kindly provided by AbbVie) on days 1, 3, 5, 8, 10, and 12. The rats were weighed on days 1, 10, 15, and 22 during the induction of vitamin D deficiency. Food consumption was measured every day. The experimental protocol was approved by the Animal Care Committee at the VU University Medical Center, Amsterdam.

### 2.2. Serum Analysis

Blood samples were drawn at days 1 and 22, respectively, before and after the initiation of the induction of vitamin D deficiency. At both time points, serum samples were analysed for 25D by competitive binding protein assay (Diasorin, Stillwater, Minnesota, USA), 1,25D by radioimmunoassay after immunoextraction (IDS, Tyne and Wear, UK), PTH by ELISA (Scantibodies Laboratory, Santee, CA), and Ca and P by colorimetric assays (Roche diagnostics, Mannheim, Germany).

### 2.3. Statistical Analysis

Serum measurements for the ten rats studied are presented as a scatter plot. As most 25D and 1,25D measurements at day 21 were below detection limits, the value of the detection level for each method was used for the statistical analysis. The majority of vitamin D levels after the intervention (both 1,25D and 25D) were below the detection limit, precluding the use of paired *t*-testing. Therefore, the nonparametric Wilcoxon signed-rank test was used to compare the differences in serum levels between days 1 and 22 using the IBM SPSS statistics 20 (Armonk, NY, US).

## 3. Results

Daily food intake was 20 gram per day (median; Inter Quartiles (IQ) 19.3–20.8) which is similar to rats fed on standard chow in terms of energy intake. In addition, there were no obvious alterations in behaviour and in the changes in body weight with time during the induction of vitamin D deficiency, compared to those observed in the same strain receiving a standard diet (data not shown).

**Figure 1:**
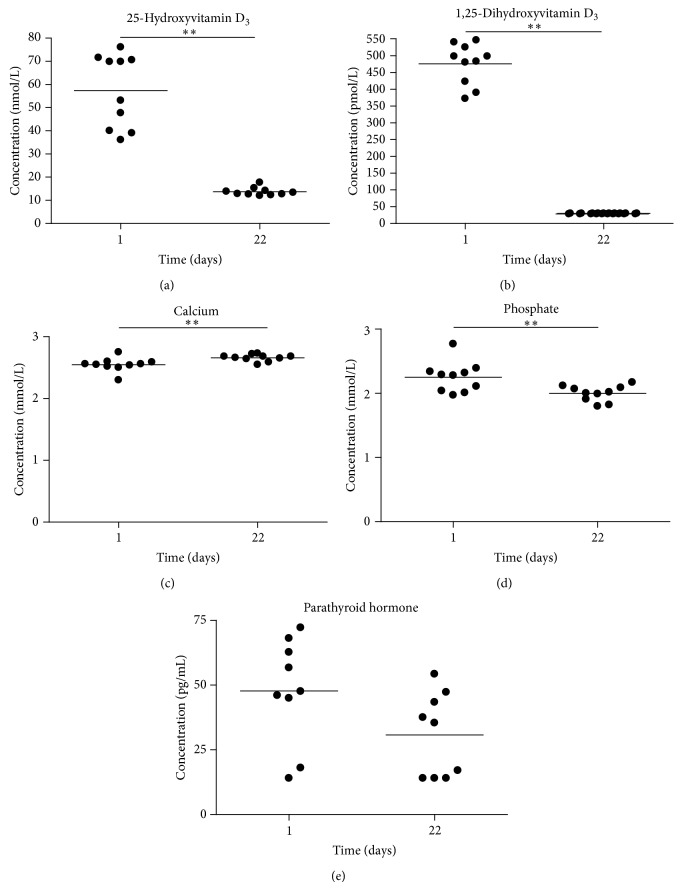
Blood chemistries at days 1 and 21 after the induction of vitamin D deficiency. Scatter plot analyses of serum levels of 25D (a), 1,25D (b), Ca (c), P (d), and PTH (e) before (day 1) and after three weeks (day 22) of starting the induction of vitamin D deficiency. Detection limits were used to depict samples which were below detection levels. The median is represented by a straight line. ^**^ *P* < 0.01 for the statistical significance of the changes in the parameter analyzed using the Wilcoxon signed-rank test.

Three weeks after the initiation of the induction of vitamin D deficiency, average 25D levels were below the detection levels (15 nmol/L) in all animals, except for three which showed a level for 25D just above detection limit (Figure 1(a)). In addition, all animals had levels below detection limit (30 pmol/L) for 1,25D (Figure 1(b)). Although the changes of serum Ca levels (increased by a median of 0.095 (IQ 0.0525–0.15) mmol/L) and serum P levels (decreased by a median decrease of 0.27 (IQ 0.0975–0.32) mmol/L) were both statistically significant (*P* < 0.01), absolute changes were mild and within normal range (Figures 1(c) and 1(d)) [20]. Moreover, importantly, there were no significant elevations in serum PTH (Figure 1(e)).

## 4. Discussion

In this study, we show that vitamin D deficiency can be safely and inexpensively induced in rats within three weeks with negligible effects on PTH, Ca, and P levels. The major advantage of this animal model is that it provides an extremely useful platform to study the unbiased effects of vitamin D deficiency and repletion, in a wide range of diseases that are currently considered to be, at least, partially driven by hypovitaminosis D. A key consideration is the undetectable levels of serum calcitriol, which in individuals with normal renal function only occur under conditions of severe vitamin D deficiency (serum 25D below 4 ng/mL) [17].

Paricalcitol injections were given exclusively during the first two weeks to induce CYP24A1 expression to cause a rapid depletion of the endogenous vitamin D metabolites. Minimal, if any, carry-over effects of paricalcitol activation of VDR biological responses in serum samples analyzed for 25D and 1,25D one week after the cessation of paricalcitol injections are expected. The rationale is that paricalcitol has a half-life of approximately seven hours in healthy subjects and fourteen hours in dialysed CKD stage 5 patients (data sheet, Zemplar). Furthermore, although in rats formal pharmacokinetic data on paricalcitol are lacking, studies on PTH suppression upon a single dose of the calcitriol analogue 22-oxacalcitriol, with a similarly short half-life to that of paricalcitol, revealed that after 96 h there was no residual action of the analogue on serum PTH but full depletion of serum calcitriol levels [21]. These reports support the chosen period of one week after cessation of paricalcitol injections to prevent carry-over effects.

Rats were fed a vitamin D depleted diet which contained high levels of Ca (2%), P (1.25%), and 20% lactose, which is commonly used to counteract the otherwise total absence of VDR-dependent intestinal Ca and P absorption of the VDR null mice, as lactose can increase the passive vitamin D-independent Ca absorption in the intestine [18, 19]. The use of a dietary Ca content (2%) higher than that in the standard rat chow (1%) was directed to both normalize serum Ca and prevent the development of hyperparathyroidism during vitamin D deficiency. To counterbalance the higher dietary Ca content, the P concentration in the diet was increased from the usual 0.7% content to 1.25% [22]. Using a similar diet, Kollenkirchen et al. showed that after six weeks, male weanling rats had normocalcemia, normophosphatemia, normal PTH levels, and 25D and 1,25D levels below 120 ng/mL and 20 pg/mL, respectively [23]. Johnson and DeLuca achieved nondetectable 25D levels with normocalcemia and a slightly decline of phosphorus levels in female rats after feeding a similar vitamin D depleted diet (2% Ca, 1.25% P, and 20% lactose) for thirteen weeks [24]. Although these studies show that hypovitaminosis D can also be induced by diet only, this requires a longer period of time, therefore increasing costs and possibly causing discomfort to the animals due to a longer exposure to abnormalities in Ca and P homeostasis.

Using the described approach, undetectable levels of both 25D and 1,25D were reached within three weeks, and importantly, normal levels of potentially confounding factors like serum PTH were maintained, along with only mild changes in serum Ca and P.

The fine adjustment of dietary Ca and P and Ca/P ratio is critical to avoid changes in Ca and P homeostasis that could invalidate any conclusion from any model of vitamin D deficiency, as shown by Fernandes et al. [15]. In fact, in rats fed a vitamin D depleted diet with 0.4% Ca and 0.3% P for 6 weeks, serum Ca levels were significantly lower while P and PTH concentrations were significantly elevated compared to control animals fed standard chow containing 0.75% Ca, 0.66% P, and 2000 IU/kg vitamin D. In contrast, rats switching from a vitamin D depleted diet with 0.4% Ca and 0.3% P to a vitamin D depleted diet with 1.6% Ca and 20% lactose, after 5 weeks, could maintain normocalcemia. However, serum PTH levels were significantly lower. P levels were significantly lower than the animals fed for six weeks on the low Ca and P vitamin D depleted diet but were not statistically different from the control animals.

Undoubtedly, although vitamin D and calcitriol deficiency will persist as long as rats are fed the vitamin D deficient diet, a three-week period is a short follow-up time to rule out the idea that signs of hyperparathyroidism or other features of prolonged vitamin D deficiency will not occur after a longer follow-up time. Whenever a longer exposure to vitamin D deficiency is necessary, it is mandatory to control for the maintenance of normal calcium, phosphate, or PTH levels, to avoid compromising result interpretation. This model, which also causes severe calcitriol deficiency, allows discriminating the impact on outcomes of the correction of active vitamin D deficiency exclusively.

## 5. Conclusion

In conclusion, we present a safe, simple, and cost effective strategy to induce severe vitamin D and calcitriol deficiency in rats without any major fluctuations in serum PTH, Ca, and P levels. Therefore, this animal model could be used to study the effects of exclusive hypovitaminosis D in many different experimental settings to mimic human disorders with only minimal potentially confounding effects of PTH and minerals. This model is a very valuable tool, not only in nephrology, but also in cancer, autoimmunity, cardiovascular, bone, and infectious disease. The precise determination of the exact role of exclusive vitamin D deficiency in all these conditions may reinforce the clinical need to adequately supplement this crucial vitamin.
